# Supplementary material for: Anatomic and hemodynamic characterization of vertebral artery duplication via color doppler ultrasonography
Source: PLoS One. 2025 Nov 14;20(11):e0336216. doi: 10.1371/journal.pone.0336216 (PMC12617842; doi:10.1371/journal.pone.0336216)
Supplement: S1 Table — (DOCX) [file pone.0336216.s001.docx]

Supplementary table 1. Descriptive statistics of 18 subjects dichotomized into vertebral artery compression syndrome group and ischemic cerebral vascular disease group

|  | Group | Number | Average | Standard deviation | Standard  deviation |
| --- | --- | --- | --- | --- | --- |
| Age | VA compression syndrome | 11 | 66.00 | 10.412 | 3.139 |
|  | ischemic cerebral vascular disease | 7 | 60.00 | 14.776 | 5.585 |
| Diameter_medial | VA compression syndrome | 11 | .2527 | .05497 | .01657 |
|  | ischemic cerebral vascular disease | 7 | .2486 | .05146 | .01945 |
| PSV_medial | VA compression syndrome | 11 | 64.373 | 23.0648 | 6.9543 |
|  | ischemic cerebral vascular disease | 6 | 60.650 | 20.8255 | 8.5020 |
| Diameter_lateral | VA compression syndrome | 11 | .2400 | .03286 | .00991 |
|  | ischemic cerebral vascular disease | 7 | .2271 | .03352 | .01267 |
| PSV_lateral | VA compression syndrome | 11 | 53.364 | 23.4221 | 7.0620 |
|  | ischemic cerebral vascular disease | 6 | 48.167 | 10.0923 | 4.1202 |
| Diameter_trunck | VA compression syndrome | 11 | .3309 | .06057 | .01826 |
|  | ischemic cerebral vascular disease | 7 | .3214 | .03625 | .01370 |
| PSV_trunck | VA compression syndrome | 11 | 48.700 | 5.7583 | 1.7362 |
|  | ischemic cerebral vascular disease | 7 | 54.757 | 12.7828 | 4.8314 |
