# Supplementary material for: Anatomic and hemodynamic characterization of vertebral artery duplication via color doppler ultrasonography
Source: PLoS One. 2025 Nov 14;20(11):e0336216. doi: 10.1371/journal.pone.0336216 (PMC12617842; doi:10.1371/journal.pone.0336216)
Supplement: S2 Table — (DOCX) [file pone.0336216.s002.docx]

Supplementary table 2. Independent-samples T test of age and ultrasound measurement parameters between vertebral artery compression syndrome group and ischemic cerebral vascular disease group

|  | | Levene homogeneity test of variance | | Student-t test for the difference in averages between groups | | | | | | |
| --- | --- | --- | --- | --- | --- | --- | --- | --- | --- | --- |
|  |  | F | Sig. | t | df | Sig. | Difference in average | Difference in standard error | 95% confidence interval | |
|  |  |  |  |  |  |  |  |  | Lower limit | Upper limit |
| Age | Assuming homogeneity of variance | .478 | .499 | 1.015 | 16 | .325 | 6.000 | 5.914 | -6.537 | 18.537 |
|  | Assuming heterogeneity of variance |  |  | .937 | 9.803 | .371 | 6.000 | 6.407 | -8.314 | 20.314 |
| Diameter of the medial limb | Assuming homogeneity of variance | .003 | .954 | .160 | 16 | .875 | .00416 | .02595 | -.05086 | .05918 |
|  | Assuming heterogeneity of variance |  |  | .163 | 13.582 | .873 | .00416 | .02555 | -.05081 | .05912 |
| PSV of the medial limb | Assuming homogeneity of variance | .028 | .869 | .328 | 15 | .747 | 3.7227 | 11.3397 | -20.4472 | 27.8926 |
|  | Assuming heterogeneity of variance |  |  | .339 | 11.381 | .741 | 3.7227 | 10.9839 | -20.3542 | 27.7996 |
| Diameter of the lateral limb | Assuming homogeneity of variance | .028 | .870 | .803 | 16 | .434 | .01286 | .01601 | -.02108 | .04680 |
|  | Assuming heterogeneity of variance |  |  | .799 | 12.727 | .439 | .01286 | .01608 | -.02197 | .04768 |
| PSV of the lateral limb | Assuming homogeneity of variance | 1.037 | .325 | .512 | 15 | .616 | 5.1970 | 10.1464 | -16.4295 | 26.8234 |
|  | Assuming heterogeneity of variance |  |  | .636 | 14.586 | .535 | 5.1970 | 8.1761 | -12.2731 | 22.6670 |
| Diameter of the trunk VA | Assuming homogeneity of variance | 1.452 | .246 | .371 | 16 | .715 | .00948 | .02552 | -.04462 | .06358 |
|  | Assuming heterogeneity of variance |  |  | .415 | 15.985 | .683 | .00948 | .02283 | -.03893 | .05789 |
| PSV of the trunk VA | Assuming homogeneity of variance | 3.419 | .083 | -1.383 | 16 | .186 | -6.0571 | 4.3782 | -15.3385 | 3.2242 |
|  | Assuming heterogeneity of variance |  |  | -1.180 | 7.574 | .274 | -6.0571 | 5.1339 | -18.0129 | 5.8986 |
